# Assessment of ventilation inhomogeneity during mechanical ventilation using a rapid-response oxygen sensor-based oxygen washout method

**DOI:** 10.1186/2197-425X-2-14

**Published:** 2014-04-16

**Authors:** Ido G Bikker, Wim Holland, Patricia Specht, Can Ince, Diederik Gommers

**Affiliations:** Department of Intensive Care, Erasmus Medical Center, PO Box 2040, Rotterdam, 3000 CA the Netherlands; Department of Experimental Medical Instrumentation, Erasmus Medical Center, Rotterdam, 3000 CA the Netherlands; Department of Anaesthesiology, Erasmus Medical Center, Rotterdam, 3000 CA the Netherlands

**Keywords:** Lung, Respiratory monitoring, Ventilatory inhomogeneity, Positive-pressure respiration

## Abstract

**Purpose:**

Ventilatory inhomogeneity indexes in critically ill mechanically ventilated patients could be of importance to optimize ventilator settings in order to reduce additional lung injury. The present study compared six inhomogeneity indexes calculated from the oxygen washout curves provided by the rapid oxygen sensor of the LUFU end-expiratory lung volume measurement system.

**Methods:**

Inhomogeneity was tested in a porcine model before and after induction of acute lung injury (ALI) at four different levels of positive end-expiratory pressure (PEEP; 15, 10, 5 and 0 cm H_2_O). The following indexes were assessed: lung clearance index (LCI), mixing ratio, Becklake index, multiple breath alveolar mixing inefficiency, moment ratio and pulmonary clearance delay.

**Results:**

LCI, mixing ratio, Becklake index and moment ratio were comparable with previous reported values and showed acceptable variation coefficients at baseline with and without ALI. Moment ratio had the highest precision, as calculated by the variation coefficients. LCI, Becklake index and moment ratio showed comparable increases in inhomogeneity during decremental PEEP steps before and after ALI.

**Conclusions:**

The advantage of the method we introduce is the combined measurement of end-expiratory lung volume (EELV) and inhomogeneity of lung ventilation with the LUFU fast-response medical-grade oxygen sensor, without the need for external tracer gases. This can be combined with conventional breathing systems. The moment ratio and LCI index appeared to be the most favourable for integration with oxygen washout curves as judged by high precision and agreement with previous reported findings. Studies are under way to evaluate the indexes in critically ill patients.

## Background

Although mechanical ventilation is critical for the survival of most patients with respiratory failure, it can also induce lung damage and may even be the primary factor in lung injury [1].

In 1970, Mead et al. estimated that forces acting on lung tissue might be 4.5 times higher when lungs are inhomogeneously ventilated [2]. This was confirmed in an experimental work using tomographic microscopy, which generates detailed three-dimensional alveolar geometries [3]. This inhomogeneity raises stress and increases the risk to develop ventilator-induced lung injury (VILI). Therefore, the use of an inhomogeneity index as a target for ventilation strategies would be very beneficial in this context.

Although inhomogeneity indexes are often used in pulmonary function labs and improve after application of positive end-expiratory pressure (PEEP) in paediatric anaesthesia, use in the intensive care unit (ICU) is limited by the need of specialized equipment and tracer gases [4, 5]. Huygen et al. [6, 7] and Gültuna et al. [8] worked on the development of inhomogeneity indexes and indicator gas injectors based on SF6 for critically ill patients, but implementation remained difficult due to the need of specialized equipment and gas containers at the bedside [6, 7]. The availability of a routine method to quantify inhomogeneous alveolar ventilation at the bedside is expected to help to optimize ventilator settings in individual patients to achieve optimal gas exchange. Recently, methods were introduced to measure end-expiratory lung volume (EELV) with an ICU ventilator based on an oxygen washout curve [9, 10]. In the LUFU system developed by Weismann et al., oxygen concentration is measured with a diverting oxygen analyser with response time < 200 ms. This method offered the possibility to develop an index of ventilatory inhomogeneity which we believe will be of great potential use in combination with EELV measurement to optimize ventilatory settings at the bedside.

The aim of this study was to develop a bedside-available computer program to compare six well-established indexes of ventilatory inhomogeneity calculated from the oxygen washout/washin curve of the LUFU system [11]. The system was evaluated for reproducibility and agreement with historical reference values in a porcine model before and after induction of acute lung injury (ALI), at four different levels of PEEP.

## Methods

The study was approved by the local animal experimental committee and was in accordance with the National Guidelines for Animal Care and Handling (permit number 142-08-01).

### Description of the indexes of ventilatory inhomogeneity

Ventilatory inhomogeneity is calculated from an oxygen washout procedure performed with the LUFU system (Dräger Medical, Lubeck, Germany). This system was developed to measure EELV based on oxygen washout and has been described earlier [12]. Briefly, O_2_ is measured with a side stream sensor, and airway flow is retrieved from the EVITA XL ventilator (Dräger Medical, Lubeck, Germany). A step change in the O_2_ concentration is induced by changing the FiO_2_. Oxygen concentration and flow are integrated in the LUFU system, and the EELV is calculated from the washout or washin procedure.

**Figure 1:**
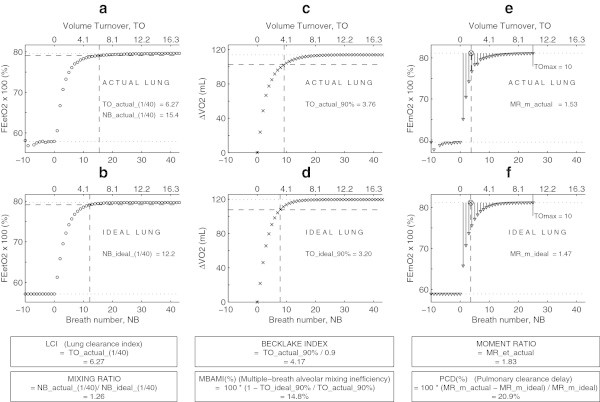
**Washin curves and calculations of the six indexes for one studied animal at 5 cm H**_**2**_ **O PEEP.** Graphs **(a, c, e)** show curves as observed from the actual lung. Graphs **(b, d, f)** show curves as calculated from an ideal lung (well-mixed one-compartment model) that has the same FRC, VD and tidal volume as the actual lung. Text boxes below the graphs contain the expressions to calculate these indexes. Horizontal axes display breath number (lower) and volume turnovers (upper). Vertical axes display expired end-tidal oxygen concentration (a, b), change in oxygen volume content in the lung (mL) (c, d) and mean expired oxygen concentration (e, f). Dotted horizontal lines represent the initial and final levels of expired end-tidal or mean concentrations (a, b, e, f) and of oxygen volume change in the lung (c, d). Dashed horizontal lines represent crossing reference lines. Dashed vertical lines determine the specific numbers of volume turnovers or number of breaths, used in the expressions to calculate the indexes.

The indexes were calculated as described in the article by Larsson et al. [11] and are shown in Figure 1. All indexes were calculated with a computer model programmed in MATLAB (MathWorks, Natick, MA, USA). Apparatus dead space was always subtracted from the tidal volume measurement when cumulative ventilation was calculated. ‘Volume turnover’ (TO) is defined as the cumulative expired volume divided by EELV. Some indexes are designed to be less dependent on tidal volume, dead space and lung volumes by comparing the measured inhomogeneity to inhomogeneity in an ideal lung. Ideal number of breaths and ideal volume turnovers refer to the washout in the simulated lung, which has the same EELV, dead space and tidal volume as the patient, but with uniform alveolar gas.

Lung clearance index (LCI) equals the number of observed volume turnovers required to reach the final end-tidal oxygen concentration within a 1/40th part of the total oxygen shift (difference between final and initial concentration levels; see Figure 1a). In Figure 1, the vertical dashed line is drawn through the intersection point of the horizontal crossing level line with the washin curve. Its upper *x*-axis coordinate value determines the needed number as TO_actual_(1/40) [13].

Mixing ratio is the ratio between the observed and the ideal number of breaths required to reach the final end-tidal oxygen concentration within a 1/40th part of the total oxygen concentration shift (see Figure 1a,b). The vertical dashed lines determine these numbers as NB_actual_(1/40) and NB_ideal_(1/40), respectively [14].

Becklake index is the number of volume turnovers required to wash 90% of the ultimate total increase of oxygen content in the lung, divided by 0.9 (see Figure 1c). The vertical dashed line is drawn through the interception point of the horizontal crossing level line with the washin curve. Its upper *x*-axis coordinate value determines the needed number as TO_actual_90% [15].

Multiple-breath alveolar mixing inefficiency (MBAMI) is defined as 100 × (1 − Volume turnover ideal/Volume turnover actual), where the number of volume turnovers refers to how many turnovers are required to wash 90% of functional residual capacity (FRC) free of tracer gas (Figure 1c,d) [16].

Moment ratio is designed to summarize the whole washout curve using moment analysis; it equals the mean residence time. It is the ratio between the first (*μ*_1_) and zeroth (*μ*_0_) moments of the washout curve [17]:

and

where *k* is the number of breaths, *η* is the number of volume turnovers at breath *k*, *N* is the number of the washout breaths at which *η*_*k*_ exceeds a preselected value (10, as mostly used), and *Y*(*k*) is the end-tidal tracer gas concentration at breath *k* minus by the concentration at the end of washin. We used the same calculation as Larsson et al. who started the summations with the first washout breath (*k* = 1). The calculated value of MR_et_actual is shown in Figure 1 upper right text box [11]. A mechanical analogon may help to understand the concept of moment ratio. Consider a mechanical balance on which a weightless beam is attached a series of weights proportional to the length of the line pieces and positioned along the beam as suggested in Figure 1e,f. The horizontal position of the pivotal point (encircled cross mark) where the beam balances determines the moment ratio.

Pulmonary clearance delay (PCD; %) is calculated as the alternative method proposed by Larsson et al., based on moment ratio: PCD (%) = 100 × (Actual moment ratio − Ideal moment ratio)/Ideal moment ratio [11]. This way of expressing PCD has the advantage of being easily adapted to automatic calculation. Contrary to Larsson et al., we truncated the summation process at TO = 10 (see Figure 1e,f). The values involved in the summation are shown as vertical solid line pieces. The involved moment ratio’s named MR_m_actual and MR_m_ideal for the actual and the ideal lung, respectively, are indicated by the drawn dashed lines.

The indexes were evaluated for integration with oxygen-based washout and automated calculation; precision was calculated as the variation coefficient. Also, values were compared with previously reported findings, also used by Larsson et al. [11].

### LUFU system

The LUFU system to measure EELV was developed and described in great detail by Weismann et al. [12]. Briefly, this system measures only oxygen with a small diverting oxygen analyser (response time < 200 ms). Currently, this system has to be connected to a Dräger ICU ventilator; in the current study, we used an Evita XL ventilator. Measurements are started by manually changing the inspired oxygen fraction with a change of at least 10%. The LUFU system then calculates the EELV from the washout/in curve, without the need for external tracer gases. Because only suitable side stream sensors are available, the resulting problem of corrupted synchronicity between flow and gas concentration measurement was solved by the use of an ingenious physical/mathematical model of the pneumatical circuit of the analyser [12, 18]. Also, the washout curve has to be meticulously corrected for oxygen consumption; the oxygen consumption is always calculated during the ten breaths before each measurement. As described by Weismann et al., measurements should be performed during steady state, with a stable cardiac output.

### Animal preparation

Seven healthy female cross-bred Yorkshire/Landrace pigs (28 to 31 ± 1.2 kg) were studied. After induction, the pigs were placed in the supine position on a thermo-controlled operation table. Anaesthesia and analgesia were maintained with an intravenous infusion with a combination of midazolam (1 to 1.7 mg/kg/h) and sufentanil (0.01 to 0.02 mg/kg/h, Sufenta Forte 0.05 mg/mL, Janssen-Cilag BV, Tilburg, the Netherlands). Muscle relaxation was obtained with an infusion of pancuronium bromide (0.17 to 0.33 mg/kg/h, Pavulon 2 mg/mL, NV Organon, Oss, the Netherlands). After tracheotomy, the pigs were connected to an EVITA XL ventilator.

In addition, an arterial catheter was inserted through the right carotid artery and a pulmonary artery thermodilution catheter (7.5 Fr, Edwards Life Sciences, Irvine, CA, USA) through the right jugular vein in the pulmonary artery. A catheter was also placed in the urinary bladder to avoid urine retention. Ventilatory data and volume-based capnography were sampled continuously during the experiment (NICO, Novametrix, Wallingford, CT, USA). Mechanical ventilation and washin and washout procedures to obtain the oxygen curves for lung volume and inhomogeneity index calculations were performed with the EVITA XL ventilator in a volume-controlled mode. Tidal volume was set at 8 mL/kg, respiratory rate was adjusted to a PaCO_2_ of 4.5 to 6.0 kPa, the inspiratory to expiratory ratio was 1:2 and PEEP was 5 cm H_2_O.

### Experimental protocol

After a stabilization period of 30 min, baseline values were recorded, and the pre-lung injury measurements were performed. Lung volume was measured with the LUFU system and was performed in quadruplicate (four washin and four washout) by changing the FiO_2_ from 0.8 to 0.6 and from 0.6 to 0.8. Washin and washout procedures were started immediately after the end of the previous procedure. After the repeatability measurements at baseline (5 cm H_2_O PEEP), inhomogeneity was measured at four decremental PEEP levels (15, 10, 5 and 0 cm H_2_O PEEP). Single measurements (one washin and one washout) were performed after a steady state of 15 min at the end of each PEEP step.

Severe ALI was induced by injection of oleic acid (0.13 ± 0.04 mL/kg, C_18_H_34_O_2_, Boom BV, Meppel, the Netherlands) into the right atrium with the PEEP set at 2 cm H_2_O. Each animal received a bolus of 0.1 mL/kg injected over 20 min to obtain a PaO_2_ below 10 kPa; if necessary, additional injections were given to reach this target [19]. After a steady state of at least 1.5 h after the induction of ALI, PEEP was increased to 5 cm H_2_O, and baseline measurements were repeated. Thereafter, the same PEEP trial was performed as described above.

### Statistical analysis

Statistical analysis was performed with the GraphPad software package (GraphPad Software Inc., San Diego, CA, USA). Results are expressed as mean ± standard deviation (SD) for normally distributed data and median ± interquartile range (IQR) for abnormally distributed data. The Shapiro-Wilk normality test was used to evaluate the distribution of all data. Changes in the inhomogeneity indexes, and hemodynamic and ventilatory parameters before and after induction of ALI, were evaluated with the Wilcoxon matched-pairs test. The Wilcoxon matched pairs-test was also used to evaluate change in the inhomogeneity indexes between PEEP steps during the decremental PEEP trial.

## Results

**Figure 2:**
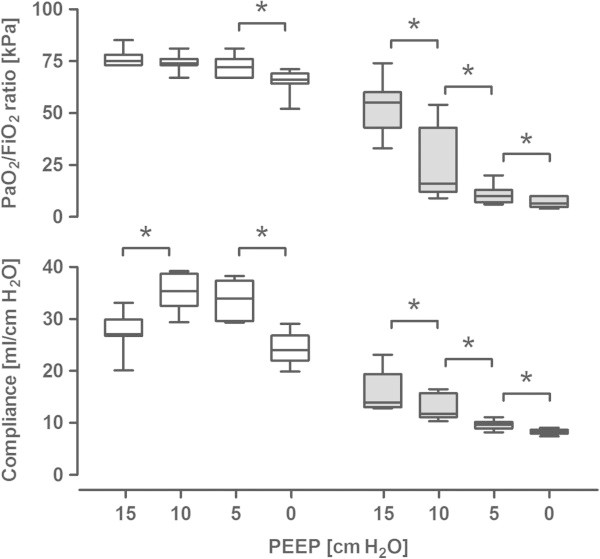
**PaO**_**2**_ **/FiO**_**2**_ **ratio and dynamic compliance at the four decremental steps in PEEP levels.** Before (open bars) and after (grey bars) the induction of acute lung injury in a porcine model. Data are presented as box-and-whisker plots (5 to 25-median - 75% to 95%). Asterisks indicate a significant difference between the PEEP levels (*p* < 0.05).

Oleic acid infusion significantly decreased EELV, arterial oxygenation and respiratory compliance (Table 1). Venous admixture and the ratio of alveolar dead space volume divided by alveolar tidal volume (VDalv/VTalv) were both increased by the induction of ALI, indicating an increased ventilation-perfusion mismatch (Table 1). Effects of the different PEEP steps on EELV, PaO_2_/FiO_2_ (PF) ratio and dynamic compliance are shown in Table 2 and Figure 2.

**Table 1:** **Data on hemodynamic and ventilatory parameters before and after induction of lung injury during baseline**

	Before acute lung injury	After acute lung injury
	(***n*** = 7)	(***n*** = 7)
Heart rate (bpm)	84 (15)	126 (29)*
MAP (mmHg)	87 (8)	88 (10)
PaCO_2_ (kPa)	4.7 (0.5)	6.4 (0.5)*
PaO_2_ (kPa)	54.2 (4.0)	8.0 (4.3)*
Shunt (%)	10 (3)	52 (19)*
VD alveolar/VT alveolar (%)	28 (5)	44 (7)*
Compliance (mL/cm H_2_O)	22 (4)	10 (2)*
EELV (L)	0.65 (0.12)	0.21 (0.02)*
Lactate (mmol/L)	0.9 (0.4)	0.9 (0.3)

Data are means ± SD. *p < 0.05.

**Table 2:** **EELV and inhomogeneity index parameters during different levels of PEEP before and after ALI**

	PEEP (cm H_2_O)							
	Before acute lung injury				After acute lung injury			
	15	10	5	0	15	10	5	0***α***
EELV (mL)	1,458	1,064*	720*	400*	848*	446*	238*	182*
Lung clearance index	6.1	6.3	6.4*	7.3*	7.2	9.8*	12.9*	19.7
Mixing ratio	1.2	1.2	1.2	1.1	1.2	1.3	1.5*	1.4
Becklake index	4.2	4.2	4.5	5.6*	4.9	6.7*	11.7*	19.3
MBAMI (%)	13.6	12.9	14.8	20.6	15.6	25.8*	45.2*	39.5
Moment ratio	1.6	1.7	1.7*	2.0*	2.0	2.4*	2.9*	3.9
Pulmonary clearance delay (%)	14.7	13.0	10.6	10.0*	15.9	17.4	20.2	10.3

Values are expressed as means. *p < 0.05, significance compared to PEEP 15 (Wilcoxon matched-pairs test), *α*; *n* = 6 (unable to perform measurements in two piglets).

The calculated six ventilation inhomogeneity indexes during baseline are shown in Table 3. The mean variation coefficients, as a measure of repeatability, are also presented (Table 3). LCI, mixing ratio, Becklake index and moment ratio were comparable with historical reference values and showed acceptable mean variation coefficients (Table 3). MBAMI and PCD were not comparable with historical reference values and showed very low repeatability (mean variation coefficient 33.5 and 29.3, respectively). PCD showed no correlation between washout and corresponding washin measurements. After induction of ALI, LCI, Becklake index, MBAMI and moment ratio significantly increased (Table 3). The slope of phase 3 of the volume-based capnogram was also significantly increased after induction of ALI (Table 3).

**Table 3:** **EELV and inhomogeneity index parameters before and after induction of lung injury during baseline**

	Before acute lung injury	After acute lung injury	Mean variation coefficient %	N_2_washout in normal subjects [11]
	(***n*** = 7)	(***n*** = 7)		
EELV (mL)	537 (127)	159 (55)**	5.2	
Lung clearance index	6.7 (0.6)	12.7 (4.3)**	10.4	7.1 (1.3)
Mixing ratio	1.2 (0.1)	1.3 (0.1)	7.8	1.57 (0.22)
Becklake index	4.6 (0.5)	11.2 (4.5)**	11.3	3.7 (0.8)
MBAMI (%)	14.6 (6.0)	35.8 (19.4)*	33.5	25 (10)
Moment ratio	1.8 (0.1)	3.0 (0.5)*	5.6	2.02 (0.14)
Pulmonary clearance delay mean (%)	11.0 (4.2)	13.7 (7.8)	29.3	31 (25)
Slope phase 3 capnogram (kPa/L)	2.1 (1.2)	7.0 (3.7)*		

All measurements were performed at 5 cm H_2_O of PEEP. Capnogram values are averaged over 100 successive breaths and increased significantly after induction of ALI. Values are expressed as mean (SD). *p < 0.05; **p < 0.01 (Wilcoxon matched-pairs test).

The inhomogeneity indexes at the different PEEP levels are shown in Table 2. Before ALI, LCI and moment ratio significantly increased after lowering PEEP from 10 to 5 cm H_2_O and from 5 to 0 (Table 2). Becklake index and PCD significantly increased after lowering PEEP from 5 to 0 before ALI (Table 2). The mixing ratio showed no response to PEEP changes before the induction of ALI. After induction of ALI, almost all indexes (except mixing ratio) increased significantly after lowering PEEP from 15 to 10 cm H_2_O, and all indexes increased significantly after lowering PEEP from 10 to 5 cm H_2_O (Table 2). At ZEEP, no significant differences were found due to a lower sample size as we were not able to measure reliably with the extensive lung injury caused by the oleic acid. MBAMI and PCD values may not be reliable due to the low repeatability as shown in the baseline measurements.

## Discussion

This paper describes the assessment and integration of a ventilatory inhomogeneity index based on a rapid oxygen sensor incorporated into LUFU equipment without the need for external tracer gases. The moment ratio and LCI index appeared to be the most favourable for integration with oxygen washout curves. To our knowledge, this is the first study to describe indexes of alveolar inhomogeneity measured by medical-grade oxygen sensors and conventional breathing systems and applicable during mechanical ventilation.

### Technical considerations

Although numerous indexes have been proposed, all require specialized equipment (e.g. mass spectrometer) and/or tracer gases (e.g. SF6, helium) [20]. To date, unfortunately, no ventilatory inhomogeneity index has been implemented for routine use in adult critically ill patients. Inhomogeneity can be determined based on single-breath or multiple-breath washout [21]. Single-breath washout is clinically available with exhaled volumetric capnography. The upslope of the third phase of this capnogram can be used as a measure of ventilatory inhomogeneity. However, a breath from vital capacity to residual volume should be used to analyse the accessible lung; during normal tidal breathing, only inhomogeneity of this ventilated volume is shown. Also, the contribution of peripheral airways is unknown. The advantage of a multiple-breath washout technique includes the evaluation of both convention- and diffusion-dependent inhomogeneity and the possibility of application during normal tidal breathing without patient cooperation. To obtain a multiple-breath washout index without the use of an insoluble external tracer gas, nitrogen and oxygen can be used as naturally available gases. Most indexes use nitrogen but require a mass spectrometer for a precise washout curve [11, 20, 22]. Oxygen washout without advanced equipment is limited by slow medical-grade oxygen sensors and the oxygen consumption correction [23]. The present study uses an improved oxygen sensor integrated in the LUFU system, originally developed and validated for EELV measurements [9, 12]. Importantly, the LUFU equipment corrects for the changing inspired oxygen fraction, which influences the gas viscosity and thereby flow through the side stream sampling system [12].

Oxygen consumption is a major factor, complicating the use of oxygen as a tracer gas. Weismann et al. developed the LUFU method for EELV measurement-based oxygen washout and previously described the calculations and basic conditions [12]. In short, oxygen transport through the lung membrane is regarded as constant throughout the washin/washout cycle. It is calculated as the oxygen consumption and tissue oxygen. During measurements, patients must be stable, with stable lung perfusion and oxygenation. Absorption is measured during stable conditions at the end of each washin or washout. This constant consumption is subtracted from each breath during washin/washout. Under these conditions, oxygen consumption does not influence these conditions.

We also implemented an automated correction for small artefacts of the washout curve in our program. The use of the fast LUFU oxygen sensor can easily lead to small disturbances caused by environmental factors (e.g. vibrations). We observed short negative disturbances in two to three successive breaths. An automated detection was implemented and correction was performed with interpolated values.

### Observed effects in the inhomogeneity indexes

We were able to calculate several inhomogeneity indexes based on oxygen washout with acceptable repeatability. At baseline, LCI, mixing ratio, Becklake index and moment ratio were comparable with historical reference values and showed acceptable variation coefficients. Moment ratio had the lowest variation coefficient, which nearly approached the EELV measurement itself. We were not able to measure inhomogeneity reliably and consistently with the PCD and MBAMI indexes. In the automatically calculated PCD, further analysis even showed no correlation between washin and washout. A possible explanation could be an attempt to compensate indexes for tidal volume, dead space and EELV by comparing the actual measured effects to the ideal effects simulated in an ideal lung (PCD, MBAMI and mixing ratio). This could exaggerate the influence of small artefacts on the calculations. The good reproducibility of the moment ratio may be explained by the underlying principle. It is not dependent on a piece of the washout curve, but rather takes the whole washout curve into account, resulting in a mean number of volume turnovers. LCI and mixing ratio are depending on the point at which the concentration is reduced to 1/40th of the initial concentration. Becklake index and MBAMI are dependent on the point where 90% of the tracer gas has been washed out and thus are heavily dependent on the tail of the washout curve. The tail of the washout curve may be especially vulnerable to artefacts when using oxygen as a soluble tracer gas.

Induction ventilatory inhomogeneity by oleic acid increases all indexes, with a significant increase in the LCI, Becklake index, MBAMI and moment ratio. This is in agreement with Tsang et al. [24]. In their study, ALI was induced with oleic acid in eight mongrel dogs, and the multiple-breath helium washout technique was used to analyse the development of ventilatory inhomogeneity. Inhomogeneity increased mainly due to an increase in the heterogeneity of tissue compliance in the peripheral airways, airway closure and a decrease in ventilation through collateral channels [24]. We also observed a significant increase in inhomogeneity using the volumetric capnogram over 100 successive breaths at baseline which evaluates breath inhomogeneity [25]. As the heterogeneity in mixing of alveolar units with different time constants increases, the slope of phase 3 of the capnogram will increase.

The LCI, Becklake index and moment ratio showed comparable increases in inhomogeneity during decremental PEEP steps before and after ALI. As shown in Figure 2, the decremental PEEP steps resulted in major reductions in PF ratio and compliance, which suggests an increased ventilation/perfusion mismatch due to inhomogeneous ventilation. The dilution factor (ratio alveolar tidal volume/EELV) could influence these results. If changed by increasing PEEP and thereby EELV, this could delay washout and increase the measured inhomogeneity. In our study, we did not find increased inhomogeneity at higher PEEP. If inhomogeneity indexes were to be used for evaluating the optimal PEEP setting, one would not only be interesting during lower PEEP steps where an increased shunt fraction can be expected. Higher PEEP may lead to lung overdistension, resulting in uneven ventilation. In our study, no increase in inhomogeneity was seen in any of the indexes. Whether these indexes are insensitive to overdistention or the used PEEP steps to low is currently unknown.

### Which index based on oxygen washout to use in critically ill patients?

In our study, LCI, mixing ratio, Becklake index and moment ratio showed acceptable precision. Inhomogeneity expressed by moment ratio analysis showed the highest reproducibility, and this index was able to detect the oleic acid-induced inhomogeneity and may be able to detect PEEP-induced changes. With its relatively low dependence on a specific part of the washout curve and thereby relatively insensitive to small artefacts, it may be favourable for oxygen washout. LCI had a slightly higher variation coefficient, but it is currently the most described inhomogeneity index in pulmonary function labs, has even been suggested as the end point for clinical trials [13] and is easy to understand. Comparing the measured values with the ideal values for an ideal lung (as used by MBAMI, PCD and mixing ratio) could theoretically improve results and reliability. In our study, this could not be confirmed; PCD and MBAMI showed low precision.

### Limitations

In the current study, we used a 20% step change in the inspired oxygen fraction, which should be feasible in most critically ill patients. Only the most seriously ill patients would require the lower step change of 10%. Also, we did not study the impact of body position; in future human studies, this might influence these measurements because off airway closure and airway secretions. Furthermore, we did use PEEP steps in a nonrandomized order which could influence results; if future PEEP titration studies would be performed, this should be a consideration.

## Conclusions

Our study has shown that ventilatory inhomogeneity can be assessed with precision, using oxygen washout curves measured with the LUFU equipment without the need for external tracer gases. The moment ratio and LCI index appeared to be the most reproducible for integration with oxygen washout curves. Studies are under way to evaluate the indexes in critically ill patients.
